# Are Oblique Views Necessary? A Review of the Clinical Value of Oblique Knee Radiographs in the Acute Setting

**DOI:** 10.5811/westjem.2022.8.56453

**Published:** 2022-10-24

**Authors:** Alexander T. Bradley, Jeremy A. Adler, Daniel M. Curtis, Darlington Nwaudo, Matthew J. Gayed, Sara J. Wallace, Aravind Athiviraham

**Affiliations:** * University of Chicago Medicine, Department of Orthopedic Surgery, Chicago, Illinois; † Stanford University, Department of Orthopedic Surgery, Palo Alto, California; ‡ University of Chicago Medicine, Department of Radiology, Chicago, Illinois

## Abstract

**Introduction:**

The purpose of this study was to assess the added clinical value of oblique knee radiographs four-view (4V) compared to orthogonal anteroposterior (AP) and lateral radiographs in a two-view (2V) series.

**Methods:**

We obtained 200 adult, 4V knee radiographs in 200 patients in the ED and randomly divided them into two groups with 100 series in each group. Ten reviewers — three musculoskeletal radiologists and seven orthopedic surgeons — performed radiograph analyses. These reviewers were randomly divided evenly into group one and group two. Reviewers were blinded to patient data and first reviewed 2V radiographs (AP/lateral) only, and then reviewed 4V radiographs, including AP/lateral, and two additional oblique views for the same patients at least four weeks later. Acute pathology identification and the need for further imaging was assessed for all reviewers, and clinical decision-making (operative vs nonoperative treatment, need for admission, need for additional imaging) was assessed only by the seven orthopaedic surgeon reviewers.

**Results:**

Mean sensitivity for pathology identification was 79% with 2V and 81% with 4V (*P* =0.25). Intra-observer kappa value was 0.81 (range 0.54–1.00). Additional oblique radiographs led orthopaedic reviewers to change their treatment recommendations in 62/329 patients (18.84%) (*P* <0.001). Eight of 329 radiographic series were identified as “critical misses.” (2.43%) (*P* =0.004), when pathology was reported as normal or reviewers recommended nonoperative treatment on 2V radiographs but changed their recommendation to operative management after the addition of oblique radiographs. The number needed to treat (NNT) for any treatment change and for “critical misses” was 83 and 643, respectively.

**Conclusion:**

Although the addition of oblique radiographs may improve a clinician’s ability to identify subtle pathologic findings not identified on 2V, it rarely leads to significant changes in treatment recommendations. Given the high NNT, limiting the usage of these oblique radiographs in the general patient population may reduce costs without significantly affecting patient care.

## INTRODUCTION

Patients commonly present to the emergency department (ED) with a chief complaint of knee pain with over 1.3 million visits each year in the US alone.^1^ These patients are often rapidly triaged, and many of them receive a radiographic examination with a four-view (4V) series, including anteroposterior (AP), lateral, and oblique radiographs as part of the initial work-up. However, there is a low overall yield for pathology identification in this population.^2^–^4^ The proposed benefit of additional oblique radiographs is an improvement in identification and classification of patellar and tibial plateau fractures.^5^ Furthermore, radiographs are generally accepted as a safe and inexpensive way to evaluate patients. However, obtaining additional radiographs beyond the two orthogonal views (AP and lateral) increases radiation exposure to patients and can lead to potentially unnecessary costs.^6^

Among ED patients who receive knee radiographs, the rate of pathology detection is low with estimates between 5.2–7.6%.^7^ Previous authors have attempted to improve the diagnostic yield of these radiographs by implementing clinical decision rules, such as the Ottawa and Pittsburgh knee rules, to minimize unnecessary radiogcraphs.^4^,^8^ However, the use of and compliance with these rule-based radiographic selection criteria are poor.^9^ Separately, the addition of oblique views as a standard component of initial knee imaging is shown to only mildly improve diagnostic sensitivity.^4^ Nevertheless, previous evaluations of oblique radiographs and clinical decision rules have focused almost exclusively on pathology identification rather than the effect of these additional imaging studies on treatment decisions.^1^,^4^–^5^,^7^–^9^ The purpose of this study was to evaluate whether the inclusion of oblique knee radiographs obtained as part of a standard 4V knee series in the emergency department (ED) setting would improve pathology identification and influence the clinical decision making for these patients.

We hypothesized that while the 4V radiographic series may improve pathology detection compared to the two-view (2V) radiographic series, the additional oblique radiographs would not lead to significant changes in clinical management, as determined by a team of orthopedic surgeons.

## METHODS

We performed a retrospective cohort study, level III evidence. Following institutional review board approval, 200 series of adult 4V knee radiographs obtained in the ED between 2010–2018 were generated from an internal radiology database using an alphanumeric report search tool and specific keywords (Appendix A). This search tool selected either positive (acute pathology) or normal (no acute pathology) radiographic series. We used an eight-year period to limit any temporal confounding from specific personnel behaviors or institutional policies that may have existed or changed over that time. Radiology reports, determined to be the reference standard, were used to determine which series contained acute pathology.

Of the 200 series there were 93 positive and 107 normal radiology reports, and these series were then randomly divided into two groups of 100 series each. The first (Group 1) included 43 positive radiographs, and the second (Group 2) included 50 positive radiographs. Five clinicians were randomly assigned to each group. Group one consisted of three orthopedic attendings or trainees and two musculoskeletal radiology attendings or fellows. Group two consisted of four orthopedic attendings or trainees and one musculoskeletal radiology fellow. While these reviewers did not include emergency physicians, we assumed that pathology identification between musculoskeletal radiologists, orthopedic surgeons, and emergency physicians should not significantly differ between groups.

**Population Health Research Capsule**

What do we already know about this issue?

*Over 1.3 million people each year present to the emergency department (ED) with knee pain and receive a four-view series of radiographs despite the low yield for pathology identification*.

What was the research question?

*Does the addition of orthogonal knee radiographs in the acute setting lead to significant changes in clinical management?*

What was the major finding of the study?

*Orthogonal radiographs slightly increase pathology identification from 79 to 81%, but rarely (NNT for critical misses 643) change operative and nonoperative treatment plans*.

How does this improve population health?

*More discretionary imaging may reduce costs without harming patient care*.

All reviewers were blinded to patient data, including clinical information, such as physical examination and history. Reviewers first evaluated AP and lateral knee radiographs (2V), and then after a time delay they reviewed AP, lateral, and oblique radiographs (4V) for the same patients. There was a minimum four-week delay between 2V and 4V analysis for all reviewers. After both 2V and 4V evaluation, all reviewers were asked to determine whether acute pathology was present and whether further imaging was required based on the available imaging only (Table 1). For orthopedic surgery reviewers only, we assessed clinical decision-making by requesting a treatment plan or management based on the radiographic findings. Management options included operative vs nonoperative treatment, hospital admission vs outpatient follow-up, or the need for additional imaging (Table 1). Using the final radiologist interpretation found in the patient’s chart, we assessed our reviewer’s abilities to accurately identify acute bony or soft tissue pathology.

We performed statistical analysis using an intra-rater reliability with Cohen kappa analysis between the 2V and 4V radiographs as well as a sensitivity analysis for each reviewer. Differences in pathology identification were assessed using Student *t*-test to detect statistically significant differences in mean values. Differences in clinical decision-making were analyzed using a one-tailed Fisher exact test. We assessed the number needed to treat (NNT) as the inverse of the absolute risk reduction from the addition of the oblique radiographs. Given the size of our study, clinical significance was set at *P* <0.05.^10^

## RESULTS

There was an average of 64.0 days (9.14 weeks) between completion of 2V and 4V analysis for the 10 reviewers. Mean sensitivity for pathology identification was 0.794 with 2V and .811 with 4V (*P* = 0.25). The intra-observer kappa value from 2V to 4V was 0.81 and ranged from 0.54 to 1 (Table 2). There were 33/1000 (3.3%) radiographic series where reviewers reported no pathology on 2V but identified acute pathology on 4V series, which we interpreted as a false negative result on the initial evaluation. Of the 33 false negatives on 2V evaluation, there were eight patella fractures (24.24%) and 16 tibial plateau fractures (48.48%). The remaining nine series were reported to have either a patellar tendon injury, tibial spine fracture, distal femur fracture, or a bone infarct.

In group one, there was a total of 43/100 positive radiograph series assessed by three orthopedic surgeons (n = 129). In group two, there were a total of 50/100 positive radiograph series assessed by four orthopedic surgeons (n = 200). In total, the seven orthopedic attendings/trainees reviewed 329 radiographic series with acute pathology, 129 in group one and 200 in group two. In 12/329 series (3.65%), reviewers recommended outpatient operative intervention based on 2V but changed their recommended clinical treatment plan to nonoperative management after the addition of oblique radiographs. There were 14/329 positive series (4.26%) where reviewers recommended further imaging after 2V but recommended nonoperative management after the addition of oblique radiographs. In 8/329 series (2.43%), the reviewer recommendation changed from nonoperative to operative management based on additional oblique radiographs (Table 3). In total, there were 62/329 series (18.84%) reviewed that experienced a change in their treatment plan after the inclusion of oblique radiographs (*P* <.001) (Table 3). Therefore, based on the general patient population knee radiograph pathology rate of 6.4%^7^, the calculated NNT for there to be a change in treatment plan was 83.

**Figure 1:**
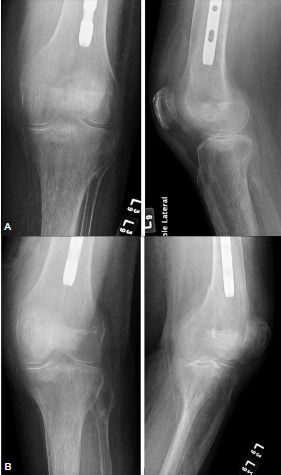
(A) Orthogonal radiographs (two-view) of one “critical miss” that was initially unidentified on orthogonal radiographs and then later identified as requiring operative management with the supplement of oblique radiographs. Seen to have a distal femoral metaphysis fracture. (B) Oblique radiographs (four-view component) of one “critical miss” that was not identified on orthogonal radiographs and then later identified as requiring operative management with the supplement of oblique radiographs. Seen to have a distal femoral metaphysis fracture.

**Figure 2:**
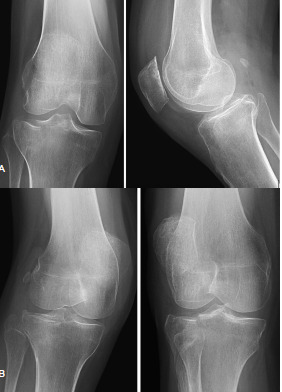
(A) Orthogonal radiographs (two-view) of one “critical miss” that was initially recommended to be best managed with nonoperative treatment but then later was recommended for operative management with the addition of oblique radiographs. Seen to have a lateral tibial plateau fracture. (B) Oblique radiographs (four-view component) of one “critical miss” that was initially recommended to be best managed with nonoperative treatment but then later recommended for operative management with the addition of oblique radiographs. Seen to have a lateral tibial plateau fracture.

**Figure 3:**
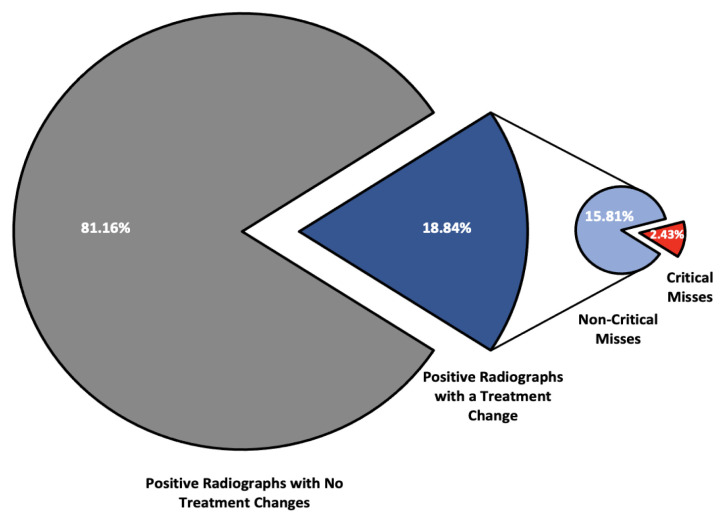
Assessment of the percentage of positive radiographs that experienced a change in treatment recommendation with the addition of oblique radiographs, including patients whose injuries were missed on initial orthogonal two-view radiographs and then went on to be recommended for surgery.

We identified patients as “critical misses” if the reviewer reported normal radiographs on 2V analysis but with the addition of oblique radiographs recommended inpatient or outpatient surgical intervention, which occurred in 4/329 (1.21%) series (Figures 1A and 1B). A “critical miss” also included series where acute pathology was correctly identified but nonoperative treatment was recommended after 2V and then transitioned to operative management after the addition of oblique radiographs. This occurred in 4/329 (1.21%) series, (Figures 2A and 2B), resulting in a total of eight “critical misses” (2.43%) in this study (Figure 3). The NNT to identify a “critical miss” was 643. For these patients, the addition of oblique radiographs significantly improved identification of “critical misses” as compared to a null hypothesis of no “critical misses” (*P* = .004).

## DISCUSSION

In this study we evaluated whether the addition of oblique radiographs can improve the efficacy of pathology identification as well as alter clinical treatment plans. Our data demonstrates a trend toward increased pathology identification with the addition of oblique radiographs, but the 4V radiographic series failed to demonstrate a statistically significant increase in sensitivity. There were 62/329 positive radiographic series that experienced a change in the expected treatment plan after reviewers evaluated the additional oblique radiographs, which had the potential to alter patient care, but only eight of those radiographic series underwent the critical transition from missed pathology to operative treatment or nonoperative to operative treatment.

Cockshott et al first discussed the benefit of additional radiographs for patients suffering knee trauma and an effusion when no acute pathology was identified on initial orthogonal radiographs.^11^ Soon after, in 1987 Daffner et al described oblique radiographs of the knee providing a more detailed evaluation of the patella by removing the projection overlap from the femur and any tibial plateau abnormalities.^12^ While additional radiographic views or tests should expectedly improve detection, the added value of these studies must be assessed against potential costs, clinical or economic, that the studies incur. Furthermore, depending on the suspected injury, there may be more beneficial imaging that targets the suspected location or type of injury, including a caudal tilt plateau view for tibial plateau fractures or an escalation to cross-sectional imaging.^13^–^15^

Despite the frequency of knee radiograph utilization in the acute setting, the positive finding rate in practice remains quite low, near 1 in 16.^7^ The value of incorporating physical examination as part of the initial evaluation prior to radiographic evaluation cannot be overstated; however, these radiographic images are often obtained prior to orthopedic surgeon or emergency physician involvement as part of the initial evaluation by a triage nurse or in an effort to expedite care. Given the low probability of acute findings, one option is to use a rule-based system to identify high-risk patients requiring radiographs in an effort to raise the yield of these studies.^16^ To this end, the Ottawa and Pittsburgh knee rules were developed as an alternative to reflexive knee radiographic imaging for all patients presenting with acute knee trauma. The Ottawa knee rules have been shown to have high sensitivity (84.6–100%, with 7 of 11 = 100%, and 9 of 11 ≥96.6%), but low specificity (19.1–52.0%, with 7 of 11 ≥41.6%)^1^,^17^–^26^ with Nichol et al demonstrating a $31–$34 savings per patient in 1999.^6^ The Pittsburgh knee rules are less widely validated but have been shown to have a similarly high sensitivity (99–100%) and improved specificity (60–80%).^8^,^24^ Despite their demonstrated value, these decision rules are not widely used during clinical decision-making.^9^,^27^ The failure to limit the number of radiographs using these rule-based approaches may stem from a fear of legal action for missed diagnoses if physicians don’t include objective evaluations such as imaging studies in their work-up.

A separate approach to limiting unnecessary radiographic imaging is to examine the effects of additional radiographic views on clinical treatment plans rather than solely evaluating pathology identification. With the addition of oblique radiographs, the percentage of patients with positive radiographs who experienced changes to their treatment plans was 18.84%. Many of these treatment plan changes would not result in delayed or ineffective care, but it is possible that a minority of patients could suffer interval fracture displacement if not properly immobilized or if given inappropriate weight-bearing instructions. If patients are told they have no injury on their radiographs, they may be less likely to follow up and this could also result in a delay of care. There were 8/329 positive radiograph series reviewed (2.43%) with more clinically important “critical misses,” including missed pathology identification (4 of 329) or inappropriate treatment plans (4 of 329), with an additional 7/349 radiographs reviewed (2.13%) transitioning from further imaging required to operative management. Given the high NNT (643), identifying these “critical misses” can require considerable resources, which should be weighed against the clinical and economic cost of failing to identify these patients on initial presentation.

While the economic cost of a single radiograph varies greatly based on hospital and location, Medicare quotes reimbursement at $112 for a single radiograph, amounting to an additional $224 for the combined oblique radiographic views per patient.^28^ Combining this with the NNT, 83 patients to identify a treatment change and 643 patients to identify a “critical miss,” these radiographs could lead to $18,592 and $144,032 in additional costs, respectively. These numbers should be evaluated from the baseline that for every single positive knee radiograph series in the acute setting, there are 15 normal radiograph series.^7^ On the contrary, there may be an economic cost not accounted for in the prior estimation from a reduction in efficiency for the additional radiographs that slows the ED workflow, specifically if a patient is initially only sent for orthogonal radiographs but then requires oblique radiographs or other imaging. But this value is hard to quantify and depends greatly on hospital-specific resources.

The cost of a delayed diagnosis or treatment for a patella fracture or tibial plateau fracture (the injuries most commonly identified on oblique radiographs) has not been well studied in the literature. Patella fractures can be treated nonoperatively when the extensor mechanism remains intact and there is minimal fracture displacement.^29^–^32^ For patients with orthogonal radiographs that do not easily demonstrate acute pathology, minimal fracture displacement would be expected, and delayed-diagnosis morbidity and cost may remain low since these patients may undergo nonoperative treatment. However, these patients often benefit from a period of temporary immobilization, which they may not receive with a missed radiographic diagnosis.^29^–^32^ This ultimately remains dependent on the specific clinician’s suspicion and examination not accounted for in this study, as the combination of radiographic studies and physical examination guides clinical decision-making.

Tibial plateau fractures may be associated with greater potential morbidity from a delayed diagnosis due to potential complications from associated soft tissue or neurovascular injuries, as well as fracture displacement potentially transitioning a nonoperative fracture to one that requires surgery. However, if a patient has a tibial plateau fracture that is difficult to visualize, it may be amenable to nonoperative treatment with bracing and limited weight-bearing.^33^–^35^ It is important to consider that the radiographs alone lack the crucial physical examination component, which aids in the diagnosis and treatment selection not included in this evaluation.

Separately, there is concern about the radiation from the additional radiographic views. A typical knee radiograph imparts 0.005 millisievert (mSv) for an adult, equivalent to nearly 1/120th of an AP pelvis radiograph or 1/1400th of a computed tomography chest (~7mSv).^36^–^37^ In another context, a flight at 35,000 feet produces 0.005 mSv radiation every hour.^37^ While it is beneficial to avoid radiation whenever possible due to its cumulative effects, the added radiation from two additional knee radiographs is minimal compared to other medical examinations that patients often undergo.

## LIMITATIONS

There are several limitations to our study that should be considered. First, this is a survey-based study of clinical experts or advanced trainees in their respective fields, but it lacked physical examination of patients, an essential component of the clinical evaluation and decision-making process. The physical examination may have offered valuable insight as to where to assess the radiographs for injury and what treatment to recommend. Second, the images provided to the reviewers were static, without the ability to alter contrast, and they may have varied in their quality of alignment without an option to obtain better radiographs, further limiting the ability to evaluate the desired anatomy. However, the quality of the radiographs obtained is often limited in the acute setting, so this may reflect normal clinical practice. Third, the evaluation was limited to a single medical center, the quality of the radiographs and radiology technicians may vary between locations, which may alter the physician’s ability to interpret the resultant imaging.

Fourth, the rate of acute pathology on our radiographs is much higher than the normal rate seen in practice. We strategically chose this higher rate of positive knee radiographs to limit the number of images the clinicians needed to review. With the inclusion of ≥50% normal radiographs for each group, we believe the integrity of radiographic assessment was maintained, as reviewers were unaware of the breakdown of positive and normal radiographs for each group. Fifth, each clinician may have slightly different clinical decision-making regarding operative and non-operative treatment, which could influence the decision for conservative or operative interventions. This should have a limited effect in this study, as changes in care based on the addition of oblique radiographs provided here were compared to each single clinician’s earlier review of the orthogonal radiographs, not to other clinicians’ evaluations. Lastly, these patients represented all patients presenting to the ED in evaluation for knee pain and were not exclusively trauma patients. While this reproduces normal workflows within our hospital, it may limit applicability of these results in certain patient populations that may have higher or lower concern for radiographically identifiable pathology.

## CONCLUSION

Oblique knee radiographs that are routinely obtained in the acute setting have been shown to potentially increase the sensitivity of pathology identification. While increased pathology identification may alter radiographically based treatment plan decision-making and affect patient care, it seldom leads to patients transitioning from nonoperative to operative management, which can have serious economic impacts. As previous studies have shown, the incorporation of a rule-based system, such as the Ottawa or Pittsburgh knee rules, may lead to a reduction in unnecessary radiographs for patients and reduce the economic burden. Given the large number needed to treat, avoiding automatic inclusion of oblique radiographs in patients may reduce costs. However, for those patients suffering a “critical miss,” it is possible they may receive delayed or inaccurate treatments counteracting these benefits. Due to the high prevalence of knee pain as a chief complaint in our acute care facilities, this topic merits a future prospective evaluation as a simple way to control the exorbitant costs faced by our patients when presenting to the emergency department.

## Supplementary Information



## Figures and Tables

**Table 1:** Survey format and questions asked for each review of two-view and four-view knee radiographs.

Survey question	Possible answers
Questions to both musculoskeletal radiologists and orthopedic surgeons	
Acute fracture or pathology identified?	1) Yes 2) No
Further imaging required?	1) CT 2) MRI 3) XRs 4) Other Imaging 5) None
Questions to only the orthopedic surgeons	
Treatment plan or management indicated?	1) Observation only 2) Nonoperative + Discharge 3) Operative + Discharge + Follow-up for Outpatient Surgery 3) Operative + Requires Admission 4) Further Imaging Required

*CT*, computed tomography; *MRI*, magnetic resonance imaging; *XR*, radiograph.

**Table 2:** Comparison of diagnostic performance.

Reviewers	Sensitivity †		Sensitivity †		Cohen intra-observer kappa statistic
	2-View	4-View	2-View	4-View	
1	0.86	0.74	0.90	1.00	0.89
2	0.86	0.81	0.90	1.00	0.83
3	0.84	0.88	0.89	1.00	0.81
4	0.91	0.91	0.93	1.00	0.98
5	0.84	0.88	0.89	0.98	0.83
6	0.84	0.86	0.85	0.96	0.68
7	0.74	0.74	0.78	1.00	0.81
8	0.68	0.86	0.75	0.86	0.54
9	0.72	0.72	0.75	0.86	1.00
10	0.66	0.69	0.75	0.96	0.78
Mean	0.79	0.81	0.84	0.96	0.81

† *P* =.251, comparing means of sensitivity.

‡ *P* <0.001, comparing means of specificity.

**Table 3:** Changes in management after the addition of oblique radiographs.

2-Views	4-Views	Number of changes	% of positive radiographs	Radiology report
Nonoperative / missed	Operative	8	2.43%	Patella fracture (4) Tibial plateau fracture (3) Distal femur fracture (1)
Nonoperative	Further imaging	9	2.74%	Tibial plateau fracture (8) Distal femur fracture (1)
Further imaging	Nonoperative	14	4.26%	Patella fracture (4) Tibial plateau fracture (1) Distal femur fracture (2) Bone infarct/lesion (2) Proximal fibula fracture (4) Segond fracture (1)
Further imaging	Operative	7	2.13%	Patella fracture (1) Patellar tendon injury (1) Tibial plateau fracture (2) Distal femur fracture (1) Proximal fibula fracture (1) Tibial spine fracture (1)
Operative (discharge and follow-up outpatient)	Operative (admit to hospital)	4	1.22%	Tibial plateau fracture (4)
Operative (admit to hospital)	Operative (discharge and follow-up outpatient)	1	0.30%	Tibial plateau fracture (1)
Operative	Further imaging	7	2.13%	Patellar tendon injury (1) Tibial plateau fracture (5) Proximal fibula fracture (1)
Operative (discharge and follow-up outpatient)	Nonoperative	12	3.65%	Patella fracture (3) Patellar tendon injury (6) Tibial plateau fracture (2) Tibial spine fracture (1)
Total number of treatment plan changes		62	18.84%†	-
Critical misses				
Missed pathology	Operative	4	1.22%	Patella fracture (2) Tibial plateau fracture (1) Distal femur fracture (1)
Nonoperative	Operative	4	1.22%	Patella fracture (2) Tibial plateau fracture (2)
Total number of critical misses		8	2.43%‡	

† *P* <.001

‡ *P* =.004
