# Supplementary material for: Are Oblique Views Necessary? A Review of the Clinical Value of Oblique Knee Radiographs in the Acute Setting
Source: West J Emerg Med. 2022 Oct 24;23(6):939–46. doi: 10.5811/westjem.2022.8.56453 (PMC9683766; doi:10.5811/westjem.2022.8.56453)
Supplement: Supplementary file 1 [file wjem-23-939-s001.docx]

Appendix A: **Radiology mPower Search Terms**

Terminology used to search our institutional radiology report database in order to identify radiographs with positive findings, and normal radiographs. After which, the radiology reports were reviewed to verify true positive or true negative results, and not incorrect initial identification.

| Positive Findings | | Normal Radiographs |
| --- | --- | --- |
| - Anatomic landmarks   - Femoral condyle   - Patella   - Tibial plateau   - Plateau   - Tibial eminence   - Eminence   - Intercondylar notch   - PCL   - ACL   - Tibial tuberosity   - Tibial spine   - Fibular head   - Fibular neck   - Fibular Styloid - Oncologic terms   - Tumor   - Lucency   - Lucent   - Lytic   - Blastic   - Cyst   - Cystic   - Malignancy | - Pathology terms   - Avulsion   - Displacement   - Displaced   - Widening   - Segond   - Hoffa   - Foreign object   - Loose body   - Loose bodies   - Arthroplasty   - Periprosthetic   - Depressed   - Depression   - Comminuted   - Transverse   - Oblique   - Stellate   - Osteochondral | - No fracture - No dislocation - Normal - No acute |
